# Spatio-Temporal Characteristics of SO_2_ across Weifang from 2008 to 2020

**DOI:** 10.3390/ijerph182212206

**Published:** 2021-11-20

**Authors:** Lining Zhu, Yu Zhang, Zheng Wu, Chengcheng Zhang

**Affiliations:** Chinese Academy of Surveying and Mapping, Beijing 100830, China; zhulining@casm.ac.cn (L.Z.); wuzheng@casm.ac.cn (Z.W.); zhangcc@casm.ac.cn (C.Z.)

**Keywords:** ground monitoring stations, SO_2_ concentration, spatio-temporal evolution, Weifang city

## Abstract

China has achieved good results in SO_2_ pollution control, but SO_2_ pollution still exists in some areas. Analyzing the spatio-temporal distribution of SO_2_ is critical for regional SO_2_ pollution prevention and control. Compared with existing air pollution studies that paid more attention to PM_2_._5_, NO_2_ and O_3_, and focused on the macro scale, this study took the small-scale Weifang city as the research area, analyzed the temporal and spatial changes in SO_2_, discussed the migration trajectory of SO_2_ pollution and explored the impact of wind on SO_2_ pollution. The results show that the average annual concentration of SO_2_ in Weifang has exhibited a downward trend in the past 13 years, showing the basic characteristics of “highest in winter, lowest in summer and slightly higher in spring and autumn”, “highest on Sunday, lowest on Thursday and gradually decreasing from Monday to Thursday” and “highest at 9 a.m., lowest at 4 p.m. and gradually increasing from midnight to 9 a.m.”. SO_2_ concentration showed obvious spatial heterogeneity: higher in the north and lower in the south. In addition, Shouguang, Changyi and Gaomi were seriously polluted. The SO_2_ pollution shifted from south to northeast. The clean wind direction (southeast wind and northeast wind) of Weifang city accounted for about 41%, and the pollution wind direction (northwest wind and west wind) accounted for about 7%. Drawing from the multi-scale analysis, vegetation, precipitation, temperature, transport situation and human activity were the most relevant factors. Limited to data collection, more quantitative research is needed to gain insight into the influence mechanism in the future.

## 1. Introduction

SO_2_ is not an air component, but it is a pollutant in the atmosphere. When a volcano erupts, it emits SO_2_. In industrial processes, the combustion of coal and petroleum containing sulfur will also generate SO_2_. SO_2_ is the main factor causing a series of environmental pollution problems such as acid rain and haze [1]. In recent decades, China has experienced accelerated industrialization and urbanization. The massive consumption of petroleum and coal energy has caused a large amount of SO_2_ emissions. As a result, China has suffered severe acid rain [2], which has also attracted the attention of the Chinese government. The Chinese government has promulgated a number of laws, regulations and policies to reduce the SO_2_ concentration. Chinese National Ambient Air Quality Standard (CNAAQS) GB3095-2012 (MEP 2012) includes two-level limits for SO_2_. The annual average concentrations for the two levels are 20 and 60 μg/m³, and the 24 h average concentrations are 50 and 150 μg/m³, respectively. The World Health Organization recommends that the 24 h average concentration of SO_2_ should not exceed 20 μg/m³.

Today, China has achieved good results in SO_2_ pollution control. However, in lower-tier cities, SO_2_ pollution still exists due to insufficient investment in environmental treatment. It is worth noting that even a very low SO_2_ concentration still has many harmful effects on human health [3,4,5,6]. It not only causes respiratory diseases, but also increases the incidence rate of lung cancer, obesity and coronary heart disease [7,8,9,10]. SO_2_ was included in the list of carcinogens published by the International Agency for Research on Cancer of the World Health Organization in 2017. In addition, many existing studies have shown that the prevalence of COVID-19 is related to SO_2_ [11,12]. For people’s health, SO_2_ pollution control in low-tier cities cannot be relaxed. The spatio-temporal analysis of SO_2_ is of great significance for low-tier cities to carry out precise prevention and control of SO_2_ pollution at low cost.

The existing research on the spatio-temporal evolution characteristics of SO_2_ mainly include research based on remote sensing inversion and research based on ground monitoring stations. The former is usually concentrated in large-scale areas, such as the whole of China [13], the North China Plain [14] and so on. The latter has the characteristics of high monitoring frequency, which facilitates better exploration of seasonal and daily changes at the urban scale (such as small- and medium-sized areas) [15,16]. In addition, ground monitoring data are much more reliable and accurate than remote sensing inversion data [17]. This paper belongs to the latter.

The existing research based on ground monitoring stations has mainly focused on large-scale areas to investigate the status and spatio-temporal variation of target air pollutants and their relationships with some factors. Mk et al. [18] used 1498 air quality monitoring points at the national scale to systematically analyze the spatial and temporal distribution characteristics of six criteria air pollutants (PM_2_._5_, PM_10_, SO_2_, CO, NO_2_ and O_3_) and their health risks. The annual average concentration of other pollutants except O_3_ is higher from the north to the south, with the highest in North China and the lowest in the Qinghai-Tibet Plateau. Maji and Sarkar [19] used hourly air quality data from more than 1000 ground monitoring stations in China, combined with air pollution control policies, to study national pollution trends in detail. Li et al. [20] selected hourly SO_2_ concentration data from 187 cities in China and found that SO_2_ is declining, with the highest in winter and the lowest in summer from 2014 to 2016. Wang et al. [21] calculated the daily average concentration of SO_2_ in five major cities of Guanzhong city, and found that the annual average concentration decreased year by year from 2014 to 2018. Rupakheti et al. [22] used the hourly concentration of six criteria air pollutants in Xinjiang from 2013 to 2019, and found the SO_2_ concentration decreased from 2015 to 2018. Most of these large-scale studies revealed the spatio-temporal evolution characteristics of SO_2_ and the impact of macroeconomic policies on SO_2_ from the macro level, but ignored small-scale spatial differences and local details.

There are also some studies based on small-scale administrative units. He et al. [23] analyzed air pollution characteristics and their relation to multi-scale meteorological conditions during 2014–2015 in 31 provincial capital cities in China. The highest rate of a major pollutant over China was PM_2_._5_ followed by PM_10_, O_3_, NO_2_, SO_2_ and CO. Meteorological conditions were the primary factor determining day-to-day variations in pollutant concentrations, explaining more than 70% of the variance of daily average pollutant concentrations over China. Kuang et al. [24] used the daily average concentration data of various pollutants from 23 ground monitoring stations in Chengdu to determine the temporal and spatial changes in pollutants and their influencing factors. The concentration of PM_10_, PM_2_._5_, SO_2_ and CO decreased from 2014 to 2016; the concentration of SO_2_ was the highest in winter and the lowest in autumn. Wang et al. [25] analyzed the spatio-temporal evolution characteristics of pollutants in urban areas of Nanjing by using the monitoring data of five stations from 2015 to 2017. The overall SO_2_ concentration at each station has an obvious downward trend. Wang et al. [26] used 13 state-controlled stations to analyze the characteristics of SO_2_ temporal and spatial changes in Xi’an from 2010 to 2018, and the SO_2_ concentration decreased significantly in eight years. Dong et al. [27] used statistics and GIS methods to analyze the air pollutants in Xiangyang city at the city and county level based on the SO_2_ concentration and other major pollutants, then discussed their characteristics, influencing factors and health effects. Wang et al. [28] used three state-controlled sites from January 2016 to February 2018 to study the characteristics of air pollution in Jiaozuo city. Lv et al. [29] used the daily average concentration of SO_2_ in Linfen city from 2016 to 2017 to explore the impact of local meteorological conditions on the concentration of air pollution. Bo [30] used the SO_2_ hourly concentration of the Harbin-Changchun area from 2013 to 2017 as the research object, discussed the current situation of pollutants, temporal and spatial changes and their relationship with meteorological factors, and found that SO_2_ concentration is related to the formation of secondary inorganic aerosols on an annual scale.

The existing SO_2_ studies based on ground monitoring stations have the following limitations. Firstly, many previous studies have tended to focus on air quality index (AQI), PM_2_._5_ and O_3_, but few have conducted in-depth studies on SO_2_. Secondly, existing research based on small-scale administrative units was mainly concentrated in developed cities of China, such as provincial capital cities, while the investment and construction of ground monitoring stations in lower-tier cities are limited, resulting in some limitations in the research on lower-tier cities, such as the following. (1) There are relatively few monitoring stations in lower-tier cities, such as 5, 8 or 13, which directly affects the accuracy of the analysis results. (2) The research time-span for lower-tier cities is relatively short, such as 1 year, 2 years and 5 years and there are only a few studies with a time span of 10 years or more, while the monitoring data with a long time-span is more reliable when analyzing the influencing factors of SO_2_ pollution such as human activities. (3) The early studies for lower-tier cities were usually based on daily data rather than hourly data, resulting in low time-resolution and insufficient granularity.

In this study, Weifang city, one of the lower-tier cities, was selected as the study area. The hourly SO_2_ monitoring data of 38 ground monitoring stations from 2008 to 2020 were used for in-depth analysis of the spatio-temporal evolution characteristics and moving footprints of SO_2_ pollution in Weifang city over the past 13 years. This study helps to enhance awareness of the temporal and spatial changes of SO_2_ in Weifang city, and also helps to provide effective prevention and control measures and targeted policy recommendations for the environmental management of Weifang city and similar areas.

## 2. Materials and Methods

### 2.1. Study Area

**Figure 1:**
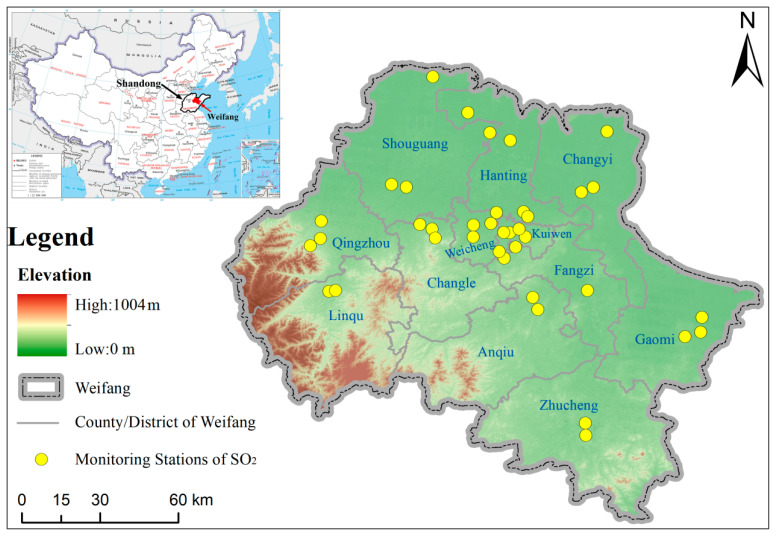
Study area and spatial distribution of monitoring stations.

Weifang (35°32′ N–37°26′ N, 118°10′ E–120°01′ E), a third-tier city, located in the central Shandong Peninsula in eastern China (Figure 1), is adjacent to Dongying in the northwest, Zibo in the west, Linyi in the south, Qingdao in the east and Laizhou Bay and the Bohai Sea in the north. It contains four districts, six cities and two counties, with a total area of 16,000 km^2^ [31]. The terrain is high in the south and low in the north. The southern and western parts are dominated by low hills, while the northeastern part consists of mainly plains, bays and rivers. Weifang is a semi-humid area with a temperate continental monsoon climate [32]. Weifang is one of the fastest-growing cities in Shandong province, with its GDP ranking the fourth in 2020. According to the ”Statistical Bulletin of Weifang city’s National Economic and Social Development in 2020”, while the economy maintains rapid growth, the environment has also been significantly improved, especially SO_2_. The amount of SO_2_ reduction in Weifang exceeded the limit of the province’s “Thirteenth Five-Year Plan”. However, there are few studies and reports on SO_2_ pollution in Weifang. Therefore, investigating and analyzing the temporal and spatial characteristics of SO_2_ are of great significance to further reduce the SO_2_ pollution in Weifang city, and can also provide a reference for other cities on how to coordinate economic development and SO_2_ pollution.

### 2.2. Data Source

The SO_2_ monitoring data used in this study were collected from the urban air monitoring network of Shandong province and the Weifang Environmental Protection Bureau in China. There are 38 automatic observation stations, including 5 national stations, 4 provincial stations and 29 urban stations. The location distribution of monitoring stations is shown in Figure 1. The data were acquired by automatic fixed air quality monitors through 24 h continuous monitoring. Thermo 43i Sulfur Dioxide (SO_2_) Analyzers were used to measure the SO_2_ concentration in the air through pulse fluorescence technology [33]. The minimum detection limit of this instrument is 1.0 ppb (60 s average time). Data are retrieved by the monitors every five minutes. All the SO_2_ data used in this paper are in units of hours. The meteorological data used in this paper from 2011 to 2020 come from a commercial weather website (https://tianqi.2345.com/ (accessed on 18 November 2021), including highest temperature, lowest temperature, weather, wind direction and wind level. The meteorological data used in this paper are in units of days.

The study period extends from January 2008 to December 2020. The invalid hourly data were filled or corrected with adjacent associated valid data. Based on the valid data above, the average SO_2_ concentrations in five time-scales (hour, day, month, quarter and year) were calculated with the arithmetic average step by step. ”Daily average” refers to the arithmetic mean of a 24 h monitoring value on a natural day. ”Monthly average” refers to the arithmetic mean of the average value of each day in a month. ”Seasonal average” refers to the arithmetic mean of the average value of each day in a season. Weifang has four seasons: spring (March, April, May), summer (June, July, August), autumn (September, October, November) and winter (December, January and February of the following year). ”Annual average” refers to the arithmetic average of the daily averages over the course of a year [34]. It is noted that the daily concentrations for SO_2_ are at least 20 h average values; the monthly concentrations for SO_2_ are at least 27 d average values (25 d average values in February); the annual concentrations for SO_2_ are at least 324 d average values [24].

### 2.3. Methodology

#### 2.3.1. Kriging Interpolation Model

Kriging interpolation can effectively identify pollution hotspots and complete the spatial distribution from the mapping surface of the entire region [35]. Many existing studies have used the Kriging interpolation model to obtain the spatial distribution map of the pollutant concentration [36,37,38]. Kriging interpolation methods can be subdivided into many kinds. The ordinary Kriging method was used to obtain the spatial distribution of SO_2_ by comparing the cross-validation results of five Kriging interpolation methods (Table 1). The comparison rules are as follows: the mean standardized value (MS) is closest to 0, the root mean square prediction error (RMS) is the smallest, the average mean error (AME) is closest to the RMS, and the root-mean-square prediction error (RMSS) is closest to 1. Given the actual size of the study area and multiple experiments conducted, we set the raster cell size to a 500 m × 500 m grid size square. The semivariogram model used for this Kriging was a spherical model. The basic formula of the ordinary Kriging interpolation model is as follows:

(1) $$\hat{Z}\left(s_{0}\right)=\overset{N}{\underset{i=1}{\sum }}\lambda_{i}Z\left(s_{i}\right)$$

where $Z\left(s_{i}\right)$ denotes the SO_2_ concentration at the observation location *i*, $\;\lambda_{i}\;$ denotes the weight of the SO_2_ concentration at the observation location *i*, $s_{0}$ denotes the predicted place and N denotes the number of observation locations.

#### 2.3.2. The SO_2_ Center of Gravity Migration Model

The SO_2_ center of gravity is the point where the SO_2_ value of all monitoring stations in the study area reaches equilibrium on the spatial plane. It can be obtained by calculating the weighted average center of gravity of all monitoring stations in the study area, where the weight value is the SO_2_ value of each monitoring station. The calculation formula of SO_2_ center of gravity is as follows:

(2) $$\{\begin{array}{c} \;\;x_{t}=\frac{\sum_{i=1}^{n}V_{i}X_{i}}{\sum_{i=1}^{n}V_{i}}\\ y_{t}=\frac{\sum_{i=1}^{n}V_{i}Y_{i}}{\sum_{i=1}^{n}V_{i}} \end{array}$$

where $x_{t},\;y_{t}\;$ represent the SO_2_ center of gravity at time *t*; *Xi*, *Yi* represent the coordinates of the monitoring station *i*; and $V_{i}$ represents the SO_2_ concentration of the monitoring station *i* at time *t*.

The SO_2_ center of gravity migration model is a model for studying the spatial changes in SO_2_ pollution based on the migration of the SO_2_ center of gravity over a period of time. It can intuitively and quantitatively reflect the direction and speed of the movement of SO_2_ pollution. The moving speed is calculated by the movement distance of the SO_2_ center of gravity. The SO_2_ center of gravity migration model  S and the moving speed ($D_{L}$) can be set as follows:

(3) $$\{\begin{array}{c} S:\{\left(\;x_{1,}y_{1}\right),\left(\;x_{2,}y_{2}\right),\cdots \left(\;x_{t,}y_{t}\right)\}\\ D_{L}=\sqrt{{\left(x_{t+1}-x_{t}\right)}^{2}+{\left(y_{t+1}-y_{t}\right)}^{2}} \end{array}$$

where $\left(x_{t,}y_{t}\right)$ denotes the SO_2_ center of gravity at time *t*. $\;D_{L}$ denotes the moving speed of the SO_2_ center of gravity from the time *t* to the time *t* + 1.

The cleaning of the raw data and the calculation of SO_2_ concentration under different time-scales (hour, day, month, season, year, etc.) were performed with C++ Language programming. The spatio-temporal interpolation, the gravity center trajectory and thematic map were made with ArcGIS 10.3 software (Environmental Systems Research Institute, Inc., Redlands, CA, USA). In addition, all statistical charts in this study were produced using Origin 9.1 software (OriginLab Corporation, Northampton, MA, USA).

#### 2.3.3. The Regression Model

The spatial difference in SO_2_ concentration in Weifang is significant and its influencing factors are diverse. This study discussed the influencing factors of SO_2_ from the perspective of meteorological factors. The linear regression equation for SO_2_ was as follows:

(4) $$Y=b_{0}+b_{1}x_{1}+b_{2}x_{2}+b_{3}x_{3}+b_{4}x_{4}+b_{5}x_{5}+\varepsilon \;$$

where *Y* denotes the SO_2_ concentration value. *x*_1_, *x*_2_, *x*_3_, *x*_4_ and *x*_5_ denote the highest temperature, lowest temperature, weather, wind direction and wind level, respectively. *b*_0_ represents the intercept; *b*_1_, *b*_2_, *b*_3_, *b*_4_ and *b*_5_ are the coefficients of the factors influencing the distribution of SO_2_ concentration and the value *ε* is a random error term. The variance expansion factor is used to analyze the influence of multicollinearity on model estimation.

## 3. Results and Discussion

### 3.1. SO_2_ Spatio-Temporal Characteristics

#### 3.1.1. Annual Spatio-Temporal Changes of SO_2_

**Figure 2:**
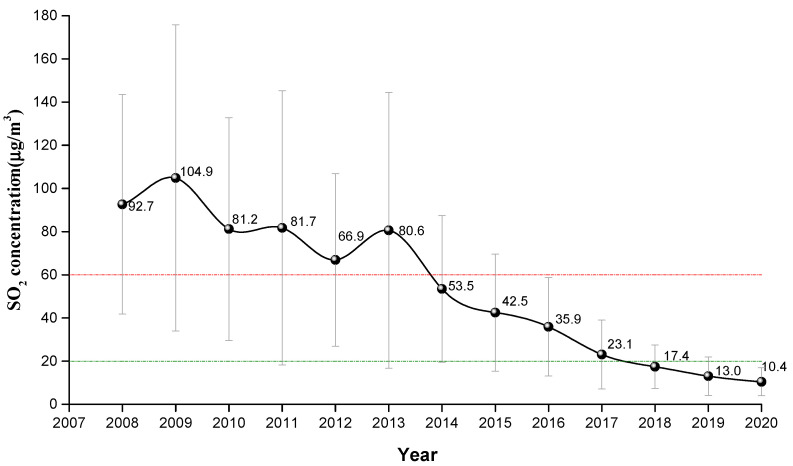
Annual average concentration of SO_2_ from 2008 to 2020. (The bars indicate average value ± S.D. The analysis was performed based on 38 observations.)

The annual average concentrations of SO_2_ for each year are shown in Figure 2. The annual average value of SO_2_ concentration in Weifang city from 2008 to 2020 showed a downward trend. The SO_2_ concentration in 2008 was about 92.7 μg/m³, while it reached its peak (104.9 μg/m³) in 2009. From 2008 to 2013, the SO_2_ concentration exceeded the CNAAQS Grade II standard (60 μg/m³). From 2014 to 2017, the SO_2_ concentration exceeded the CNAAQS Grade I standard (20 μg/m³). Since 2018, the SO_2_ concentration has met the CNAAQS Grade I. By 2020, the SO_2_ concentration was about 10.4 μg/m³. During 2008 to 2020, the SO_2_ concentration decreased by about 82.3 μg/m³. In addition, from 2008 to 2013, the SO_2_ concentration decreased in fluctuation, with a slow decline rate of about 2.42 μg/year. After 2013, the fluctuation in SO_2_ concentration disappeared and showed a continuous downward trend, with a decline rate of about 10.03 μg/year.

In Figure 2, the fluctuation of SO_2_ concentration from 2008 to 2013 may be related to the supervision of pollution control, which at times is strong and at other times is weak. In other words, it is the result of the mutual game between economic development and ecological environment protection before the successful transformation of industry in Weifang. Before industrial upgrading and transformation, Weifang was still dominated by traditional industries with high energy consumption and high pollution, such as the chemical industry and papermaking. Air pollution control had a great impact on local economic development and financial revenue. Some high-polluting enterprises started production when pollution control supervision was relaxed. Since 2013, the fluctuation disappeared. This is attributed to the fact that, under the call of national policies [39,40,41], Weifang city adhered to the principle of absolute priority for ecology, organized and implemented the “386” environmental protection action, and took law enforcement and supervision as the guarantee. The strict emission reduction measures adopted by Weifang city included the elimination of polluting vehicles, the elimination of small coal-fired boilers, the installation of exhaust gas monitoring equipment, the delineation of combustion zones, the closure of polluting enterprises, and the prohibition of the burning of straw, etc. Since then, Weifang city has achieved remarkable results in air pollution control. Affected by the COVID-19 epidemic that broke out at the end of 2019 [42,43], the annual average concentration of SO_2_ in Weifang has been significantly lower than 20 μg/m³ in 2020.

**Figure 3:**
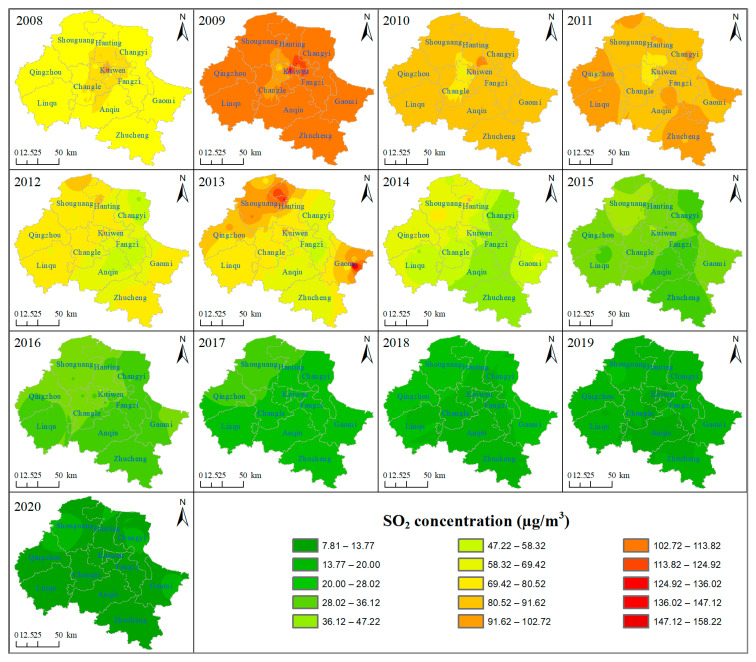
Spatial distribution of SO_2_ concentration in Weifang City, from 2008 to 2020.

Figure 3 illustrates the spatial distribution of the annual average concentrations of SO_2_ from 2008 to 2020 in Weifang. The data used for Kriging interpolation in Figure 3 are the annual average values of each monitoring station. As shown in the maps, in 2008, SO_2_ pollution was mainly concentrated in urban areas. Since 2009, the polluted areas shifted from the center to the periphery; especially in 2013, two heavily polluted areas (Hanting and Gaomi) were formed in the urban fringe. By 2020, while the overall SO_2_ concentration in the region continued to decrease, three sub-regions with poor performance had been formed in Shouguang, Changyi and Gaomi. From 2016, SO_2_ concentration featured high in the northwest and low in the southeast. There are two main reasons. (1) It is related to SO_2_ emission. According to the conclusions of Guo et al. [8] and Wang et al. [44], they believe that population density is positively correlated with industrial production scale and energy consumption, resulting in the increase in SO_2_ emission. The population and GDP scale of the western region (such as Shouguang and Qingzhou county) and the eastern region (such as Gaomi) rank among the top five in Weifang city, which shows that these regions have large SO_2_ emissions. (2) To a large extent, it is related to the terrain of Weifang city. The south of Weifang is mostly low mountains and hills with high vegetation coverage, which can effectively absorb pollutants in the air and reduce SO_2_ concentration. However, the northwestern part of Weifang is flat and adjacent to Dongying city, a traditional industrial city with more serious air pollution. It is easy for large-scale diffusion of SO_2_ to occur from Dongying city to these relatively flat areas.

#### 3.1.2. Seasonal and Monthly Spatio-Temporal Changes in SO_2_

**Figure 4:**
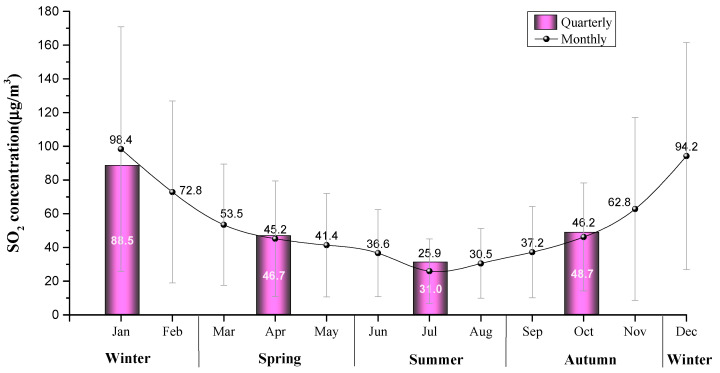
Seasonal and monthly average SO_2_ concentration from 2008 to 2020. (The bars indicate average value ± S.D. The analysis was performed based on 38 observations.)

Figure 4 shows the seasonal and monthly changes in SO_2_ concentration in Weifang from 2008 to 2020. The monthly curve was obtained by averaging monthly observations from 2008 to 2020, and the seasonal value was obtained by averaging seasonal observations from 2008 to 2020. The monthly average SO_2_ concentration formed a U-shaped trend. The typical U-shaped inflection point appeared in July (25.9 µg/m³), which means the SO_2_ concentration in July was the lowest in the whole year. The SO_2_ concentration fell from January to July, while it rose from August to December. The SO_2_ concentration was higher in January (98.4 µg/m³) and December (94.2 µg/m³).

As shown in Figure 4, the variation in SO_2_ concentration in Weifang city has obvious seasonality. It is characterized by the highest in winter, followed by autumn and spring, and the lowest in summer. From 2008 to 2020, the average SO_2_ concentrations in spring, summer, autumn and winter were 46.7 µg/m^3^, 31.0 µg/m^3^, 48.7 µg/m^3^ and 88.5 µg/m^3^, respectively. The concentration difference between winter and summer was about 57.5 µg/m^3^.

The terrain and winter meteorological conditions lead to high levels of pollution in winter in Weifang. Typically, low temperature, weak airflow and low rainfall in winter are not conducive to diffusion of pollutants, and have become important natural factors for serious pollution in winter [45]. The northwest wind prevails in winter and the hilly terrain in the south is not conducive to the diffusion of pollutants. In addition, due to the cold weather, coal-fired heating causes a large amount of SO_2_ emissions [46] and the exhaust gas emitted by private cars contains SO_2_ [47], which triggers the increase in SO_2_ concentration. Conversely, in summer, high temperature and strong airflow that favor diffusion make air pollution levels lower, and high rainfall and humidity are prone to pollutant dilution and wet deposition. The southeast wind prevails in summer and the flat northwest region accelerates the diffusion of pollutants. In spring and autumn, air pollution is exacerbated by crop straw burning [48,49,50], producing intermediate levels of pollution.

**Figure 5:**
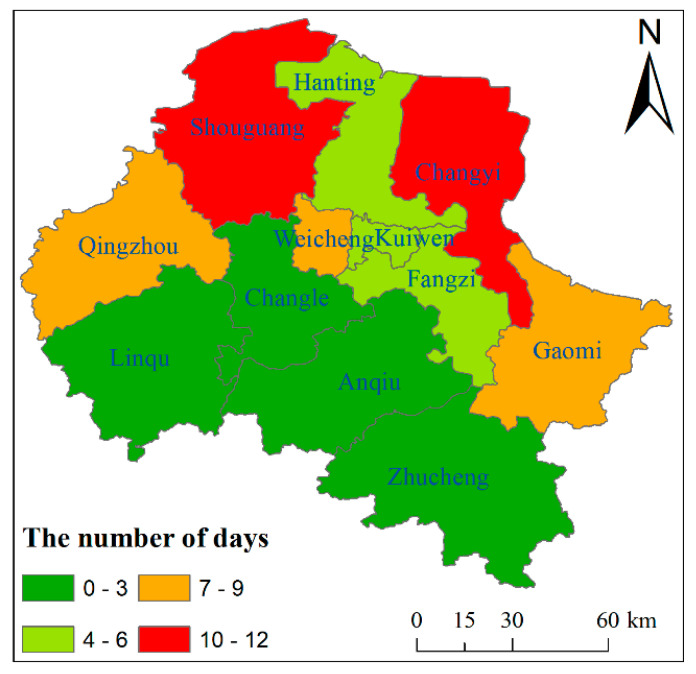
The days of daily average concentration of SO_2_ exceeding 20 μg/m^3^ in winter of 2020 in Weifang.

The World Health Organization recommends that the daily average concentration of SO_2_ should not exceed 20 µg/m^3^. Since SO_2_ pollution was the most serious in winter, the number of days in which the SO_2_ average daily concentration exceeded 20 μg/m^3^ in the winter of 2020 was counted using the regional statistical method. The result is shown in Figure 5. It can be seen from Figure 5 that the SO_2_ average daily concentration exceeding 20 μg/m^3^ occurred more frequently in the northern region than in the southern region. Specifically, Shouguang and Changyi were the highest, followed by Qingzhou, Weicheng and Gaomi; Hanting, Kuiwen and Fangzi were lower; Linqu, Changle, Anqiu and Zhucheng were the lowest. Although the concentration of SO_2_ in Weifang was decreasing year by year, the daily average concentration of SO_2_ in winter exceeding 20 μg/m^3^ still exits, especially in the north.

#### 3.1.3. Weekly Spatio-Temporal Changes in SO_2_

**Figure 6:**
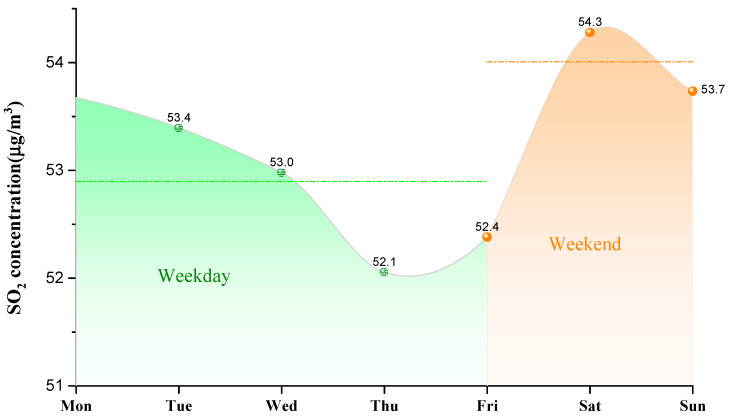
Daily average concentration of SO_2_ during a week from 2008 to 2020.

As shown in Figure 6, the daily average concentration of SO_2_ during a week in Weifang city presented a periodic S-shaped curve fluctuation law from 2008 to 2020. The SO_2_ concentration was higher on Monday (53.7 μg/m^3^) and Saturday (54.3 μg/m^3^), and lower on Thursday (52.1 μg/m^3^). It has a significant “weekend effect” [51]. The reasons for this result are as follows.

After work on Friday, many people go out to relax and welcome the upcoming weekend. Increased human activities increase the SO_2_ concentration. On Saturday, many people go out for recreational purposes, or visit relatives and friends, which results in excessive human and transportation activities, leading to increasing the SO_2_ concentration. On Sunday, some people choose to stay at home, the number of people going out decreases, and thus the SO_2_ concentration drops slightly. On Monday, when people return to work, human activities lessen, therefore the SO_2_ concentration continues to decrease, reaching the lowest on Thursday. By comparing the SO_2_ concentration on weekdays (from Monday to Friday) and the weekend (Saturday and Sunday), it can be seen that the SO_2_ concentration is closely related to human behavior, work and rest.

**Figure 7:**
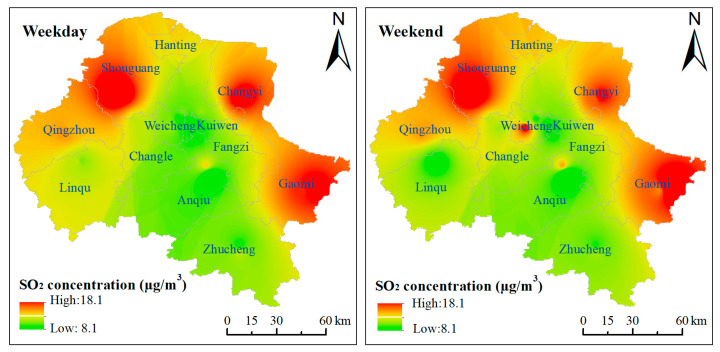
Comparison of the spatial distribution of SO_2_ on weekdays and weekends in Weifang City in 2020.

Figure 7 shows the spatial distribution of SO_2_ pollution on weekdays and the weekend in 2020. The data used in the Kriging interpolation are the daily average concentrations at each monitoring station on weekdays and weekends during 2008–2020. It can be seen from Figure 7 that the SO_2_ concentrations in Shouguang, Changyi and Gaomi were higher than in other regions both on weekdays and weekends, which was also consistent with the overall spatial variation in 2020 (Figure 3). In addition, the SO_2_ concentration in Weicheng district increased significantly on rest days. The reason may be that as a central district, Weicheng district is more prosperous in culture, tourism and shopping, which attracts people to visit on weekends, resulting in the increase in SO_2_ concentration.

#### 3.1.4. Hourly Changes in SO_2_

**Figure 8:**
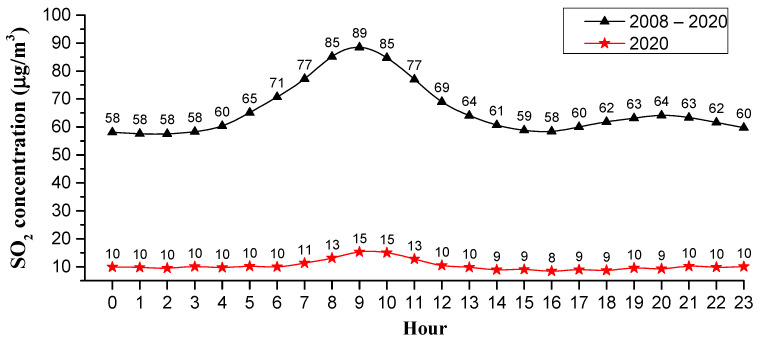
Hourly average concentration of SO_2_ from 2008 to 2020.

Figure 8 shows the hourly change in SO_2_ concentration in Weifang city from 2008 to 2020. In Figure 8, the triangles are the average hourly concentrations during 2008–2020, while the stars are the average hourly concentrations in 2020. The hourly average peak value of SO_2_ concentration was 89 µg/m^3^ at 9 o’clock and the valley value was 58 µg/m^3^ at 16 o’clock, with a difference of 31 µg/m^3^.

**Figure 9:**
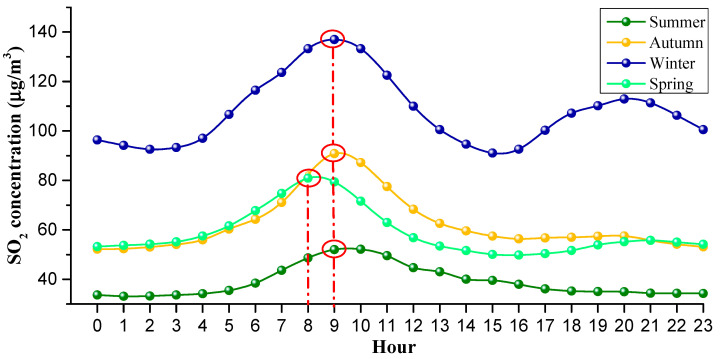
Hourly average concentration of SO_2_ in different seasons from 2008 to 2020. The circles mean the local maximum points in different seasons and the dotted lines describe the vertical lines corresponding to the circles.

The hourly SO_2_ concentration changes in different seasons, and the peak of hourly SO_2_ concentration varied with the seasons (see Figure 9). Specifically, the morning peak in the SO_2_ concentration in spring occurred at 8 a.m., while the morning peak in SO_2_ concentration in summer, autumn and winter occurred at around 9 a.m. This may be due to the temperature on spring mornings being very comfortable and people being inclined to get up early, thereby the morning peak for work and school came earlier, which caused the morning peak in SO_2_ concentration in spring to be one hour earlier. It is noted that the SO_2_ concentration exhibited an evening peak in winter that occurred at about 8 p.m., while in other seasons, there was no significant evening peak. After 4 p.m., parents pick up their children from school, and people gradually finish work, ushering in the evening peak of off-work and school. In winter, due to the cold weather, people often take cars when they finish work or pick up their children from school; the heavy traffic causes the rise in the SO_2_ concentration. However, in other seasons except the winter, the night temperature is comfortable, especially in summer and autumn; people prefer to walk or ride a bicycle when they finish work or pick up their children, thus the SO_2_ concentration has not lifted significantly during the evening rush hours. The hourly changes in SO_2_ in Weifang city are basically consistent with residents’ commuting activities, indicating that automobile exhaust is one of the main sources of SO_2_ [52].

### 3.2. The SO_2_ Center of Gravity Migration Trajectory

**Figure 10:**
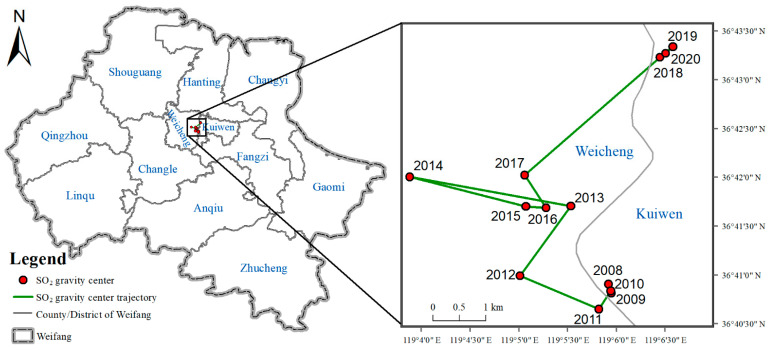
SO_2_ center of gravity from 2008 to 2020.

The trajectory of the SO_2_ center of gravity from 2008 to 2020 is shown in Figure 10. It can be seen that in the north-south direction, the SO_2_ center of gravity shifted northward as a whole, indicating that the SO_2_ pollution in the south was gradually reduced. The north-south migration can be divided into three stages: 2008–2012 is the first stage, 2013–2017 is the second stage and 2018–2020 is the third stage. In the east-west direction, the SO_2_ center of gravity first shifted westward and then eastward. Specifically, from 2008 to 2014, it mainly shifted to the west, and from 2014 to 2020, it mainly shifted to the east.

On the whole, from 2008 to 2020, the SO_2_ center of gravity shifted to the northeast. Especially in 2017-2018, the shift to the northeast was obvious, with an offset distance of about 3 km, indicating that the SO_2_ pollution in the northeast region is more serious than other regions. This may be related to the rapid development of the Binhai Economic Development Zone located in the northeast coastal area after 2017.

### 3.3. The Correlation Analysis between SO_2_ and Wind

The regression model results are shown in Table 2. Since the highest temperature and lowest temperature did not pass the collinearity test, we eliminated the lowest temperature. As shown in Table 2, wind direction and wind level have a significant impact on SO_2_ concentration. The wind direction in the model is negative (−7.49454) and the wind level is positive (6.97515).

**Figure 11:**
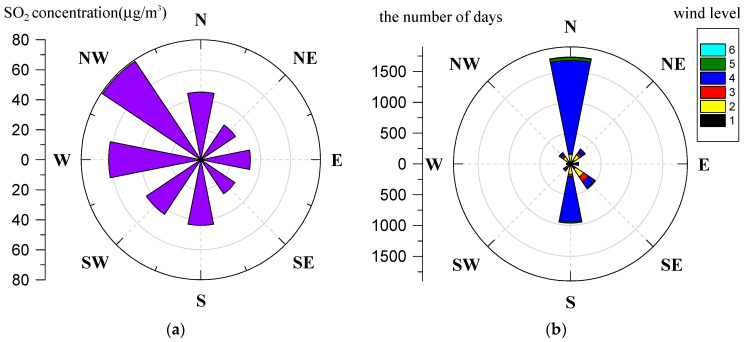
(**a**) Relationship between SO_2_ and wind direction; (**b**) frequency map of wind direction.

The regression model results were consistent with Chen et al. [53], who discovered that the main meteorological driving factor of pollutant concentration in North China is wind. Figure 11a shows the relationship between SO_2_ concentration and wind direction, where SO_2_ concentration was the overall average value from 2011 to 2020. The radius represents the concentration; the larger the radius, the higher the concentration. As can be seen from Figure 11a, when the wind blew from the west, the SO_2_ concentration in Weifang was high, and when the wind blew from the east, the SO_2_ concentration in Weifang was low. According to the wind direction, the SO_2_ concentration was northwest wind > west wind > north wind > south wind > southwest wind > east wind > northeast wind > southeast wind. When the northwest wind and westerly wind prevailed, the SO_2_ concentration in Weifang city was obviously higher, indicating that the northwest wind and westerly wind have a polluting effect on the SO_2_ in Weifang city. When the southeast wind and northeast wind prevailed, the SO_2_ concentration in Weifang city was significantly lower, indicating that the southeast wind and northeast wind have a cleaning effect on the SO_2_ in Weifang city. The reasons are as follows. (1) The terrain in the west of Weifang is the highest, which hinders the wind from the west, resulting in the weakening of the wind. The northeast of Weifang is close to the Bay, and the weak wind from the west has difficulty dispersing the pollutants to the sea area. (2) When northwest wind and west wind prevail, Weifang is probably in winter; the temperature is low, which is not conducive to diffusion, and there are many SO_2_ emission sources such as coal-fired heating. When the southeast wind prevails, Weifang is probably in summer, and the temperature in summer is high, which is conducive to the diffusion of pollutants.

Figure 11b shows the frequency map of the wind direction in Weifang from 2011 to 2020. The north wind accounted for the highest proportion, about 43%, and the south wind followed with about 24%. In addition, the clean wind direction (southeast wind, northeast wind) accounted for about 19%, while the polluted wind direction (northwest wind, west wind) accounted for about 7%.

## 4. Conclusions

This paper presented the temporal and spatial characteristics of SO_2_ pollution on different time scales (yearly, seasonal, monthly, daily and hourly) based on data collected from the hourly state-controlled and provincial-controlled ground monitoring stations in Weifang city. Furthermore, the trajectory of SO_2_ pollution and the relationship between the concentration of SO_2_ and meteorological factors were discussed. Based on the above study, the following conclusions were mainly drawn:

(1) The average concentration of SO_2_ showed a decreasing trend from 2008 (92.7 ± 50.9 µg/m^3^) to 2020 (10.4 ± 6.5 µg/m^3^). Before 2014, the SO_2_ concentration showed a fluctuating downward trend, and exceeded the CAAQS Grade II standard (60 µg/m^3^). Since 2014, the SO_2_ concentration has been continuously decreasing. In 2018, SO_2_ concentration was lower than the CAAQS Grade I standard (20 µg/m^3^). This contributed to the strict measures taken by the Chinese government, and the environmental protective actions implemented by the Weifang city government, such as elimination of polluting vehicles, elimination of small coal-fired boilers, installation of exhaust gas monitoring equipment, delineation of no-burning zones and so on.
(2) The spatial pattern of SO_2_ pollution shows that the SO_2_ concentration presented obvious spatial heterogeneity. Specifically, the SO_2_ concentration was higher in the north than in the south, and higher in the west than in the east. In 2008, the SO_2_ concentration in central city was significantly higher than that in surrounding areas. Since 2009, the SO_2_ pollution shifted from the urban center to the outside. By 2013, two heavily polluted areas (Hanting and Gaomi) were formed in the marginal area. At present, Shouguang, Changyi and Gaomi have the most serious SO_2_ pollution. We recommend optimizing the industrial structure of these three regions to control the SO_2_ pollution.
(3) On the seasonal scale, the SO_2_ concentration was winter > autumn > spring > summer. In the winter of 2020, the daily average concentration of SO_2_ in the northern part of Weifang was still exceeding 20 µg/m^3^, while the World Health Organization recommends that the daily average concentration of SO_2_ should not exceed 20 µg/m^3^. The reason for this phenomenon was the weather conditions in winter and coal-fired heating. It is recommended that relevant departments in Weifang take measures to reduce the SO_2_ concentration in the northern region in winter, such as central heating, natural gas heating and polluting enterprises to temporarily suspend production.
(4) On the daily scale, the SO_2_ concentration on the weekend was higher than that on weekdays, and the SO_2_ concentration was highest on Saturday. On the weekend, the SO_2_ concentration in Weicheng district, the central district of Weifang, increased significantly. The daily-scale variation characteristics of SO_2_ concentration are closely related to human travel and work. It is recommended that Weifang citizens travel greener and more staggered on the weekend.
(5) On the hourly scale, the peak in the SO_2_ concentration was around 9 a.m. and the valley was around 4 p.m. The peak value varied with the seasons. In detail, the peak in spring occurred at 8 a.m., while the peak in other seasons occurred around 9 a.m. This phenomenon shows that hourly scale changes are closely related to residents’ commuting activities. In Weifang city, it is possible to implement staggered commuting and promote new energy vehicles. In addition, we can also vigorously develop public transportation to avoid excessive SO_2_ concentration in the short term, which threatens people’s health.
(6) The SO_2_ center of gravity migrated to the northeast as a whole. This shows that the SO_2_ pollution in the northeast of Weifang is more serious than other areas, especially from 2017 to 2018. This result may be related to the construction of the Binhai Economic Development Zone located in the northeast coastal area. We can reduce the SO_2_ pollution in these areas by optimizing the industrial model of the Binhai Economic Development Zone.
(7) From the correlation analysis between SO_2_ and wind, the order of the SO_2_ concentration from high to low is as follows: northwest wind > west wind > north wind > south wind > southwest wind > east wind > northeast wind > southeast wind. The clean wind direction (southeast wind, northeast wind) accounted for about 19%, while the polluted wind direction (northwest wind, west wind) accounted for about 7%.

This research has important practical significance for in-depth understanding of the temporal and spatial changes of SO_2_ in Weifang city since 2008. As a pollutant that successfully achieves emission reduction, studying its temporal and spatial characteristics has momentous reference value for the subsequent treatment of SO_2_ pollution and the environmental treatment of other pollutants. This paper mainly focused on the temporal and spatial characteristics of SO_2_ pollution at the multi-scales. In future work, the correlation between air pollution and multi-source factors will be analyzed in depth, including natural and human influences.

## Figures and Tables

**Table 1:** The cross-validation results of five Kriging interpolation methods.

Kriging Method	MS	RMS	AME	RMSS
Ordinary	−0.00081	0.00794	0.00901	0.98858
Simple	−0.02663	0.00797	0.00784	1.11777
Indicator	0.00069	0.42417	0.46007	0.93451
Probability	0.00104	0.43544	0.45898	0.96046
Disjunctive	0.00365	0.00786	0.00756	1.05141

MS: the mean standardized value; RMS: the root mean square prediction error; AME: the average mean error; RMSS: the root-mean-square prediction error.

**Table 2:** The results of multiple regression.

	Estimate	Std. Error	t	*p* (>|t|)
Intercept	92.20235	3.32988	27.689	0.00
Highest temperature	−1.70441	0.05003	−34.07	0.00
Weather	−1.66185	0.30851	−5.387	0.00
Wind direction	−7.49454	0.48851	−15.342	0.00
Wind level	6.97515	0.6012	11.602	0.00

## Data Availability

The air quality data can be collected from the urban air monitoring network of Shandong province. The website is http://fb.sdem.org.cn:8801/AirDeploy.Web/AirQuality/MapMain.aspx (accessed on 18 November 2021). The meteorological data can be obtained from the commercial weather website at https://tianqi.2345.com/ (accessed on 18 November 2021).
